# Knee arthroscopy and exercise versus exercise only for chronic patellofemoral pain syndrome: a randomized controlled trial

**DOI:** 10.1186/1741-7015-5-38

**Published:** 2007-12-13

**Authors:** Jyrki A Kettunen, Arsi Harilainen, Jerker Sandelin, Dietrich Schlenzka, Kalevi Hietaniemi, Seppo Seitsalo, Antti Malmivaara, Urho M Kujala

**Affiliations:** 1 The ORTON Research Institute, Invalid Foundation, Tenholantie 10, FIN-00280 Helsinki, Finland; 2 The ORTON Orthopaedic Hospital, Invalid Foundation, Tenholantie 10 FIN-00280 Helsinki, Finland; 3 The Helsinki University Central Hospital, Jorvi Hospital, Turuntie 150, FIN-02740 Espoo, Finland; 4 The Finnish Office for Health Care Technology Assessment/National Research and Development Centre for Welfare and Health, Lintulahdenkuja 4, FIN-00530 Helsinki, Finland; 5 The Department of Health Sciences, University of Jyväskylä, P.O. Box 35, FIN-40014 University of Jyväskylä, Finland

## Abstract

**Background:**

Arthroscopy is often used to treat patients with chronic patellofemoral pain syndrome (PFPS). As there is a lack of evidence, we conducted a randomized controlled trial to study the efficacy of arthroscopy in patients with chronic PFPS.

**Methods:**

A total of 56 patients with chronic PFPS were randomized into two treatment groups: an *arthroscopy group *(*N *= 28), treated with knee arthroscopy and an 8-week home exercise program, and a *control group *(*N *= 28), treated with the 8-week home exercise program only. The arthroscopy included finding-specific surgical procedures according to current recommendations. The primary outcome was the Kujala score on patellofemoral pain and function at 9 months following randomization. Secondary outcomes were visual analog scales (VASs) to assess activity-related symptoms. We also estimated the direct healthcare costs.

**Results:**

Both groups showed marked improvement during the follow-up. The mean improvement in the Kujala score was 12.9 (95% confidence interval (CI) 8.2–17.6) in the arthroscopy group and 11.4 (95% CI 6.9–15.8) in the control group. However, there was no difference between the groups in mean improvement in the Kujala score (group difference 1.1 (95% CI -7.4 - 5.2)) or in any of the VAS scores. Total direct healthcare costs in the arthroscopy group were estimated to exceed on average those of the control group by €901 per patient (*p *< 0.001).

**Conclusion:**

In this controlled trial involving patients with chronic PFPS, the outcome when arthroscopy was used in addition to a home exercise program was no better than when the home exercise program was used alone.

**Trial registration:**

Current Controlled Trials ISRCTN 41800323

## Background

Patellofemoral pain syndrome (PFPS) is a common problem and has an impact on many aspects of daily life [1,2]. The possibly multifactorial etiology of PFPS is partially unknown and a wide range of conservative and surgical procedures has been used to treat patients with the syndrome [3].

There is some evidence that exercise therapy reduces anterior knee pain in patients with PFPS [4]. Although many physicians prefer conservative therapy modalities in the treatment of PFPS with unknown origin of pain, in chronic cases and after the failure of conservative treatment, arthroscopy is often carried out. However, although a few randomized controlled trials (RCTs) have compared the effects of various operative techniques [5,6], no RCTs either analyzing the diagnostic value of arthroscopy or comparing surgical interventions with conservative therapy in the treatment of PFPS have been published. Surgical interventions in PFPS should be based on diagnostic findings suspected to underlie the pain syndrome [2]. Arthroscopy is used to make a specific diagnosis and to perform finding-specific surgical procedures. To further clarify the additional value of diagnostic and operative arthroscopy, we conducted a randomized trial to assess the efficacy of arthroscopy in conjunction with a home exercise program (arthroscopy group) versus home exercise program only (control group) in patients with chronic PFPS.

## Methods

### Study participants

Orthopedic surgeons identified consecutive female or male PFPS patients who had been admitted to either the ORTON Orthopaedic Hospital, Helsinki, or one of the outpatient clinics of the public hospitals of the Hospital District of Helsinki and Uusimaa between May 2003 and February 2005. According to our study protocol 56 patients were needed to detect a clinically significant difference between the outcomes of the groups studied. All of the patients who fulfilled the first-stage selection criteria (Table 1) and gave their preliminary consent to participate in the study attended a structured clinical interview and examination held in the ORTON Orthopaedic Hospital and carried out by an experienced orthopedic surgeon (DS) who was not involved in the treatment of these patients. The patients also answered a structured questionnaire. If the patient did not fulfill the final (second stage) inclusion criteria (Table 1) they were excluded. On the basis of the orthopedic surgeon's examination and knee X-ray findings four patients did not fulfill the final inclusion criteria and were excluded from the study. Three of them had symptoms and signs of meniscal injury and one patient had a disabling general illness. Furthermore, one patient refused to participate before randomization. Among patients with bilateral knee symptoms, the knee with the more severe symptoms was included in the study. All of the 56 patients who fulfilled the final inclusion criteria signed an informed consent immediately after the clinical examination and before randomization.

**Table 1:** Inclusion and exclusion criteria among patients with PFPS

The first stage (evaluation in the ORTON Orthopaedic Hospital or in the outpatient clinics of the public hospitals in the Helsinki area)	
Inclusion criteria	Exclusion criteria
Age 18 – 40 years	Disabling general illness
Female or male	Reported knee ligamentous or meniscal injuries
Characteristic history of PFPS and symptoms lasting at least 6 months	Previous knee surgery
Patellofemoral pain during knee loading physical activity, such as jumping, running, squatting, or going up or down stairs	Physician diagnosed knee osteoarthritis
Patellofemoral pain when the knee was kept in flexion for a prolonged period, with relief on extension	A history of patellar dislocation; however, subjects with patellar subluxation are included in the study
	Other knee problems than PFPS diagnosed clinically (such as jumper's knee)
	Other knee problems than PFPS diagnosed radiographically (such as osteochondritis dissecans)
	Physical therapy for PFPS within the previous 4 weeks
	Pregnancy
	Competitive athlete

The second stage (orthopedic surgeon's clinical and radiological evaluation in ORTON)	
Inclusion criteria	Exclusion criteria

As in stage one	Clear medio-lateral instability in manual instability measurement
	Knee problems other than PFPS
	Knee osteoarthritis
	Osteochondritis dissecans, loose bodies in the patellofemoral and tibiofemoral joints

### Randomization process and treatment groups

**Figure 1:**
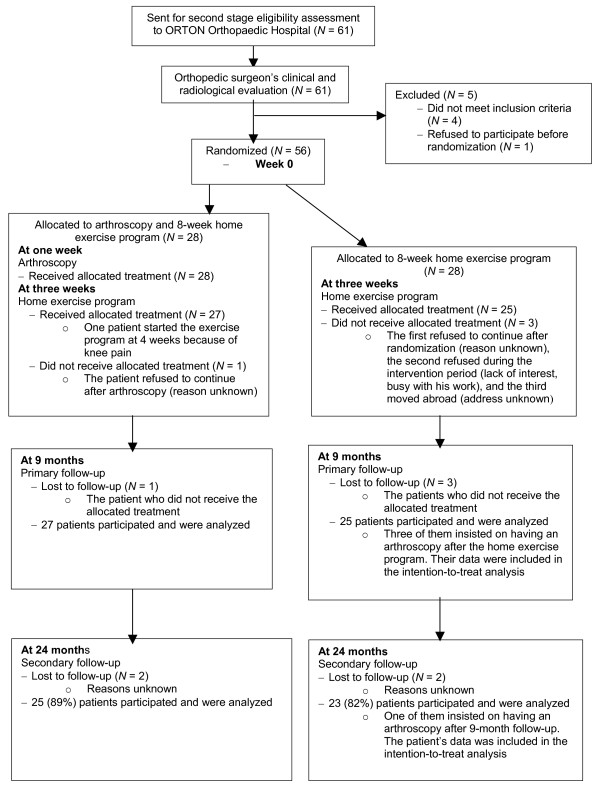
Trial profile.

The patients were randomized into two treatment groups: an *arthroscopy group*, which was treated with knee arthroscopy and 8-week home exercise program, and a *control group*, which was treated with the same 8-week home exercise program only (Figure 1). The randomization process was carried out using a computer-generated randomization list stratified by gender. Sealed, sequentially numbered envelopes containing information on the treatment group were prepared and given to the assisting nurse, who opened the envelopes in numerical order after recruitment so that concealment of allocation was successful in all cases.

### Exercise protocol

The 8-week home exercise program (see Additional File 1) was started 3 weeks after randomization in all patients (2 weeks after arthroscopy in the arthroscopy group). At the first visit an experienced physiotherapist gave instruction individually on lower-limb muscle strengthening and stretching exercises to be performed daily during the first four weeks at home. The approximate duration of each session was 30 min. The second visit was during the third week from the beginning of the exercise period. For the resisted knee extension and flexion exercises in the second part of the program, all of the patients were given a rubber sling to be used around the ankle. Again, the patients were instructed to repeat the prescribed exercises daily. They were instructed to avoid symptom-producing activities during the intervention. The duration of each daily home exercise session was approximately 30 min.

### Arthroscopy

All of the patients who were randomized into the arthroscopy group received arthroscopy; however, one of them refused to continue treatment thereafter. Arthroscopy was performed one week after randomization by one of the two experienced knee orthopedic surgeons (AH, JS). All of the knee compartments were examined systematically and pathological findings were recorded. The stage of cartilage lesions in the patellofemoral joint was recorded on a standard form according to the Outerbridge [7] classification. During arthroscopy the following procedures were performed, if justified on the basis of the arthroscopic findings and according to our pre-determined guidelines, which followed generally accepted recommendations [2]: resection of inflamed/scarred medial plicae, abrasion of chondral lesions and shaving of excessive and inflamed synovium. Minor corrections of the patellofemoral articulation were performed, such as lateral capsular discision in the case of clear lateral patellar subluxation in the beginning of knee flexion. Moreover, possible meniscal tears were treated. These patients remained in the study group and they participated in the standardized training protocol. One patient, however, was unable to start the exercise program at 2 weeks following the arthroscopy because of pain, and the start of her training was adjusted individually.

### Outcome measures

Outcome measures were collected using self-administered questionnaires. This data collection was organized by the study coordinator (JAK). As the coordinator did not have any presuppositions as to which of the groups would show better results and because he was not a treatment provider, the data collector was not, for practical reasons, blinded to the treatment groups. The measurements were completed before randomization, immediately after the end of home training period and 9 months after randomization. At each time point the patients filled in the questionnaire alone in a quiet room. The patient-completed questionnaire was then checked by the study coordinator and missing items, if any, were answered. The 24-month evaluation was conducted using a postal questionnaire. The primary outcome measure was the Kujala score (see Additional File 2) [8], also known as the anterior knee pain score [9], which is a 13-item questionnaire including different items on pain related to functioning and activities. The categories within each item are weighted and item scores summed to provide an overall index scored from 0 to 100 where the maximum score of 100 represents no disability. Other investigators have shown that the questionnaire is a reliable, valid and responsive outcome measure for PFPS [9], patients have described it as easy to understand and as depicting the symptoms well [10], and the score has been used widely in evaluating patellofemoral disorders in scientific studies. According to Crossley et al [9] a clinically significant improvement is deemed to have occurred when the patient's score shows an increase of around 8–10 points.

As secondary outcome measures we used three 10-cm visual analog scales (VASs) to assess activity-related pain. Participants assessed the maximum pain they had felt during the previous 2 days when ascending stairs, descending stairs and standing up from a sitting position. Also, a global rating of change between baseline and follow-up (overall assessment) was evaluated with an additional six-point scale: 1, asymptomatic knee, to 6, marked worsening. The scale was later dichotomized and analyzed as follows: (1) marked worsening, moderate worsening or no change from baseline versus (2) moderate improvement, marked improvement or asymptomatic knee.

### Diary

The patients kept a diary during the therapy intervention. The patients calculated the weekly frequency with which they had followed the exercise protocol. The diary also included questions on pain and discomfort (VAS), possible complications and use of drugs as well as other healthcare services and treatments not related to the study protocol. The patients were advised to avoid other therapies during the exercise period.

### Economic evaluation

Direct healthcare costs included the baseline orthopedic surgeon's visit, the costs of the interventions and additional visits to healthcare providers during the intervention and follow-up period [11].

### Data analysis

The statistical analysis was done with Statistical Package for the Social Sciences 15.0 (Norusis/SPSS, Inc., Chicago, IL). The sample size was calculated to detect a mean difference of 10 points in the Kujala score between the treatment groups, using a standard deviation of 13 determined on the basis of our pilot material [12]. To obtain a less than 5% probability of a type-I error and a power of 80%, 27 participants were required in each group. We randomized 56 patients, allocating 28 to each study group.

The primary analysis was intention-to-treat and the primary follow-up time was 9 months from randomization. Comparisons between the groups were performed with analysis of covariance using the baseline scores as a covariate. The secondary follow-up time was 24 months from randomization. In addition, we carried out 'a worst-case scenario' analysis of the data. In this analysis, we assumed that the Kujala score would have been the same as the baseline score (no change), if follow-up data were not available.

### Ethical approval

The ethical committee of the Hospital District of Helsinki and Uusimaa and the review board of the ORTON Orthopaedic Hospital approved the study protocol.

## Results

Fifty-six patients underwent randomization (Figure 1). The baseline characteristics were similar between the groups (Table 2). Also, no group difference was observed in the reported work-related physical loading at baseline. Compliance with the training protocol was similar in both study groups: mean weekly exercise frequency was 5.0 in the arthroscopy group and 5.2 in the control group (*p *= 0.52).

**Table 2:** Baseline characteristics of the randomized patients

	Arthroscopy group (*N *= 28)	Control group (*N *= 28)
Characteristics		
Age, years	28.4 (7.5)	28.4 (5.6)
Female % (*N*)	60.7 (17)	64.3 (18)
Height, cm	171.7 (10.2)	172.4 (9.6)
Weight, kg	69.0 (19.3)	71.4 (15.1)
BMI, kg m^-2^	24.1 (3.3)	23.8 (3.6)
Duration of symptoms, months	54.9 (73.4)	45.0 (74.9)

There were no statistically significant differences between the groups.

Data are mean (SD) or per cent (*N*).

## Nine-month follow-up

One patient in the arthroscopy group and three in the control group were lost to the follow-up (Figure 1). In the arthroscopy group 37% (10/27) and in the control group 20% (5/25) of the patients reported that they had used oral anti-inflammatory analgesics during the follow-up, either during the home training period or afterwards (*p *= 0.23).

Three patients in the control group insisted on having an arthroscopy after the exercise intervention and prior to the 9-month follow-up. The arthroscopy findings of these patients were normal knee, softening of the patellar cartilage and marginal medial meniscus rupture. These patients participated in the follow-up and were analyzed according to their original group assignment. The absolute changes in the Kujala score in these patients were 7, 13 and -4, while among the remaining patients in the control group the mean improvement was 12.7.

The mean improvement in the Kujala score between randomization and follow-up was 12.9 (95% confidence interval (CI) 8.2–17.6, *p *< 0.001)) in the arthroscopy group and 11.4 (95% CI 6.9–15.8, *p *< 0.001) in the control group. No difference between the two groups was observed in mean improvement according to the Kujala score or VAS scores (Table 3). Similar results were obtained from the 'worst-case scenario' analysis (mean difference in Kujala score 2.0; 95% CI -8.0 - 4.1, *p *= 0.523)).

**Table 3:** Results of intention-to-treat analysis of outcomes by group

	Arthroscopy group		Control group		
	At baseline	9-month follow-up	At baseline	9-month follow-up	Difference in mean change scores (95% CI)^1,2^
Kujala score, mean (SD)	69.0 (10.7)	81.9 (14.1)	71.1 (13.0)	82.5 (15.3)	1.1 (-7.4 to 5.2)
Pain when descending stairs, VAS mean (SD)	43.4 (27.2)	21.1 (23.0)	35.0 (26.9)	18.0 (23.3)	0.9 (-10.1 to 11.9)
Pain when ascending stairs, VAS mean (SD)	48.8 (29.9)	20.9 (24.7)	41.1 (29.4)	20.5 (24.9)	2.6 (-10.0 to 15.2)
Pain when standing up from a sitting position, VAS mean (SD)	39.0 (28.1)	16.6 (22.4)	41.4 (28.7)	21.8 (25.3)	4.1 (-7.0 to 15.2)

^1^The difference is positive, when the mean change in score was greater for arthroscopy than for control group.

^2^Adjusted for baseline values.

In the arthroscopy group 82% (22/27) and in the control group 76% (19/25) of the patients reported at least moderate improvement at the end of the follow-up period (*p *= 0.74). When the arthroscopy and control groups were combined, the mean change in the Kujala score among those patients who reported no improvement during the 9-month follow-up was 5.0 (SD 10.5) and among those with at least moderate improvement it was 14.1 (SD 10.8), *p *= 0.016.

### Arthroscopy findings and outcome

The arthroscopy findings and surgical procedures in the arthroscopy group are shown in Table 4. The Kujala score improved between randomization and follow-up in the arthroscopy group in those whose arthroscopy findings were normal (*N *= 5) by 17.0 points and in those with at least one abnormality documented at arthroscopy (*N *= 22) by 12.0 points (Table 4). The Kujala score improved by 12.8 points among the 17 patients who had at least one abnormality and who had surgical procedures owing to their abnormalities, and by 9 points among those who had abnormalities, but did not have any surgical procedure (*N *= 5).

**Table 4:** Arthroscopy findings and treatment procedures among patients in the arthroscopy group

			Kujala score		
			
Arthroscopy findings	Number of arthroscopy findings in 28 knees	Treatment procedures	at baseline, mean (SD)	at 9-month follow-up, mean (SD)	mean change (95% CI)
Articular cartilage lesion; no other findings					
Grade I	1	No treatment	52.0	58.0	6.0
Grade II	4	No treatment	68.0 (11.1)	86.3 (11.0)^1^	14.8 (9.5 to 20.0)^1^
Grade III	8	Shaving	66.1 (13.0)	82.0 (12.5)	15.9 (8.7 to 23.0)
Grade IV	1	Shaving	51.0	59.0	8.0
Inflamed/scarred plicae; no other findings	4	Resection	73.0 (10.1)	79.8 (8.8)	6.8 (-11.5 to 25.0)
Meniscal lesion; no other findings	1	Partial resection	72.0	52.0	-20.0
Patellar subluxation; no other findings	1	No treatment	71.0	66.0	-5.0
Patellofemoral dysplasia; no other findings	1	Lateral capsular discision	74.0	98.0	24.0
Articular cartilage lesion and inflamed/scarred plicae	1	Shaving and plica partial resection	66.0	93.0	27.0
Articular cartilage lesion and meniscal lesion	1	Shaving and meniscal partial resection	74.0	100.0	26.0
Normal findings	5	No treatment	72.2 (11.7)	89.2 (9.5)	17.0 (1.6 to 32.4)

^1^N = 3; one patient lost to 9-month follow-up

### Costs and use of healthcare services

Table 5 shows the mean direct healthcare costs and use of healthcare services per patient for PFPS during the intervention and the follow-up period. The mean difference per patient in total direct healthcare costs between the groups was €901 (95% CI 714 – 1095, *p *< 0.001). The mean number of sick leave days was 8.7 in the arthroscopy group and 1.4 in the control group (*p *< 0.001).

**Table 5:** Costs and use of healthcare services among patients with PFPS in the arthroscopy and control groups during the intervention and follow-up period

		Number of visits, mean per patient		Direct healthcare costs (€), mean per patient	
Type of utilization	Cost/visit (€)	Arthroscopy group	Control group	Arthroscopy group	Control group
Medical specialist care	174.50	1.18	1.29	205.90	225.10
Knee arthroscopy	1039.00	1.00	0.11	1039.00	114.30
Physiotherapy (session of 30 minutes maximum)	33.50	2.11	2.25	70.70	75.40

Total direct healthcare costs (€), mean per patient				1315.60	414.80

The number of visits includes baseline visit by the orthopedic surgeon and number of visits for the allocated intervention (arthroscopy and physiotherapy).

### Twenty-four-month follow-up

Twenty-five (89%) patients from the arthroscopy group and 23 (82%) patients from the control group answered the 24-month follow-up questionnaire. The mean improvement according to the Kujala score was maintained at the 24-month follow-up in both the arthroscopy group and control group (mean scores 12.5 and 9.4, respectively). No difference in the mean change in the Kujala score between randomization and the 24-month follow-up was found (baseline-adjusted difference arthroscopy versus controls was 2.8; 95% CI -4.2 – 9.9, *p *= 0.42).

## Discussion

Our study shows that arthroscopy did not provide any overall additional advantage for chronic PFPS patients when provided in addition to the training program. Our economic analysis showed that the direct healthcare costs were higher in the arthroscopy group compared with the control group. Moreover, the higher numbers of days on sick leave among the patients in the arthroscopy group indicate that the indirect healthcare costs were also higher in the arthroscopy group.

Our arthroscopy procedure was based on recommendations on the etiology and arthroscopic treatment of PFPS [2]. Despite a statistically insignificant trend towards a baseline difference between the groups in the Kujala score, it is likely that abnormalities in the patellofemoral joint were evenly distributed between our arthroscopy and control groups. Furthermore, when investigating the effects of treatment between the groups, we adjusted our results with the baseline values. Also, in the arthroscopy group, the patients with abnormalities who were operated on using recommended surgical procedures did not show greater improvement than the other patients.

Two patients were shown to have meniscal pathology in arthroscopy. However, both of these patients reported anterior knee pain symptoms at the baseline examination. It is known that some patients with meniscus problems report anterior knee pain symptoms, but we cannot be sure whether these symptoms were in fact due to meniscus pathology in our subjects. These patients were treated according our preliminary pre-determined guidelines, which followed generally accepted recommendations.

We do not know whether the number of abnormalities seen in our arthroscopy group is higher than might be found in a non-symptomatic population. Interestingly, the mean improvement was somewhat better among the patients whose arthroscopic findings were normal. The mechanism responsible for the effectiveness of the treatment among these patients is unknown. Patients with chronic pain expect aggressive treatment and a patient's expectations may influence the clinical outcome independently of the treatment itself [13]. Arthroscopy itself may have a placebo effect [14]. This possible placebo effect does not change the conclusions drawn from our study.

Only patients with symptoms of at least 6 months duration were included in our study and after the follow-up one-fifth of these patients reported no improvement compared with the baseline situation. Although PFPS is usually a non-progrediating condition, our result is in line with earlier findings that, despite adequate treatment, some patients with PFPS have long-term symptoms [15]. Although different stage cartilage lesions seem to be quite common among patients with PFPS, the association between cartilage lesion and symptoms is usually considered to be weak [1,16]. In our previous study on PFPS patients, only severe, possibly pre-arthrotic, cartilage injury predicted persistent long-term symptoms [12]. In the present study only one patient had a stage IV cartilage lesion and his Kujala score improved by 8 points.

While our primary aim was to investigate the possible additional benefit of knee arthroscopy among patients with PFPS, an exercise program consisting of simple lower-limb stretching and strengthening exercises was used as a generally accepted treatment to improve the adherence to our arthroscopy versus no-arthroscopy study design. As our patients had experienced prolonged knee symptoms, it would have been difficult and perhaps unethical to use a no-treatment control group. Also, the number of withdrawals and dropouts would have been higher among patients without any treatment. Although three patients from the control group insisted on an arthroscopy during the follow-up, their outcome was no better than that reported by the other controls.

The fairly high mean weekly exercise frequency indicated good treatment compliance in both groups. A speculative, alternative interpretation of our results is that exercise was effective both with and without the addition of arthroscopy. It should be emphasized that the primary aim was not to investigate the effectiveness of exercise therapy in patients with PFPS. In fact, we found no association between frequency of weekly exercise and improvement (data not shown). Despite that, our results are in line with the earlier view that exercise therapy may be an effective treatment for PFPS [4].

Competitive athletes, who may have had pain syndrome related to extreme loading and perhaps not be willing to follow the present treatment protocols, were excluded from the study. Also, patients younger than 18 and those over 40 years of age were excluded. Our results are valid for young adults who do not participate in competitive sports. Our study shows that an unselected group of chronic PFPS patients received no benefit from arthroscopy in addition to exercise therapy. The study does not show whether there are any subgroups of PFPS patients who would benefit from arthroscopy. However, this is possible.

Although our primary end-point was at 9 months, as we wished to avoid many of the random factors, which can influence longer follow-ups, we also collected data at 24 months. Extending the follow-up did not change the result.

In conclusion, on the basis of our randomized study, which was planned using available knowledge on the diagnosis and methods of treating PFPS, arthroscopy cannot be recommended for patients with chronic PFPS.

## Competing interests

The author(s) declare that they have no competing interests.

## Authors' contributions

JAK participated in all parts of the study, including the design and coordination of the study, data collection and writing of the manuscript. DS performed the baseline clinical examinations and participated in the design of the study, data collection and writing of the manuscript. AH and JS performed the arthroscopies and participated in the design of the study, data collection and writing of the manuscript. KH participated in the design of the study, data collection and writing of the manuscript. SS participated in the design of the study, data collection and writing of the manuscript. AM contributed to planning the study and writing the manuscript. UMK was the research leader in charge, and he participated in the design of the study, obtaining funding, data analysis and writing of the manuscript. All of the authors saw and approved the final manuscript.

## Pre-publication history

The pre-publication history for this paper can be accessed here:



## Supplementary Material

Additional File 1 The 8-week home exercise program for patients with PFPS. Click here for file

Additional File 2 The Kujala score. Click here for file
