# Supplementary material for: Knee arthroscopy and exercise versus exercise only for chronic patellofemoral pain syndrome: a randomized controlled trial
Source: BMC Med. 2007 Dec 13;5:38. doi: 10.1186/1741-7015-5-38 (PMC2249589; doi:10.1186/1741-7015-5-38)
Supplement: Additional File 1 — The 8-week home exercise program for patients with PFPS. [file 1741-7015-5-38-S1.pdf]

PFPS Exercise protocol, both arthroscopy and control group

First part of the program (weeks 1-4)

First two exercise weeks:

- Exercise every day and repeat the protocol twice daily.
- Exercises numbers 1 to 4: 10 – 20 repetitions each.
- Exercises numbers 5 to 7: 3 – 5 repetitions each.

Weeks three and four:

- Exercise every day and repeat the protocol four times a day.
- Exercises numbers 1 to 4: 10 – 40 repetitions each.
- Exercises numbers 5 to 7: 3 –5 repetitions each.

Second part of the program (weeks 5-8)

Weeks five and six:

- Exercise every day and repeat the protocol twice daily.
- Exercises numbers 8 to 11: 10 – 20 repetitions each.
- Exercises numbers 12 to 14: 3 –5 repetitions each.

Weeks seven and eight:

- Exercise every day and repeat the protocol four times a day.
- Exercises numbers 8 to 11: 10 – 40 repetitions each.
- Exercises numbers 12 to 14: 3 –5 repetitions each.

Home exercise program both arthroscopy and control group

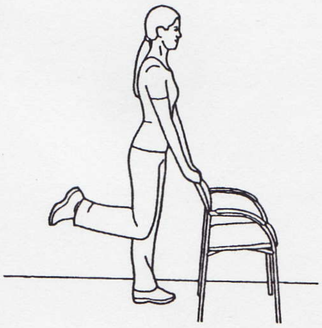

Exercise number 1.

Stand. Hold onto a support and bring one leg slightly backwards.

Bend your knee and lift your foot off the floor. Hold it for a little while and slowly let it come down. You should be able to feel the tension in the back part of your thigh.

© PhysioTools Ltd

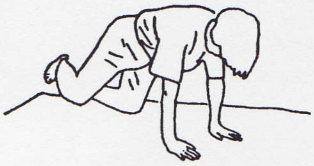

Exercise number 2.

On all fours, lift the leg you are exercising sideward with the knee bent, and let it come down. You should be able to feel the tension in your buttock.

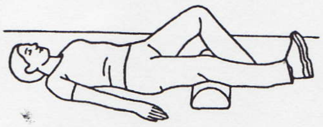

Exercise number 3.

Lie on your back with a cushion under the thigh of the leg you are training.

Pull your foot up, tighten your thigh muscles and straighten the knee. Hold the tension for a while and then relax. Repeat. Keep your thigh on the cushion during the exercise.

© PhysioTools Ltd

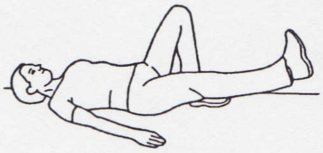

Exercise number 4.

Lie on your back with one leg straight and the other leg bent.

Exercise your straight leg by pointing your foot upwards and tightening your thigh muscles. Twist your leg outwards and lift it up and down.

© PhysioTools Ltd

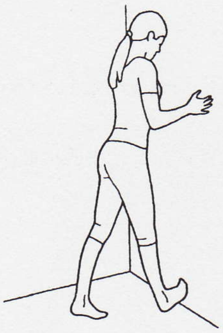

© PhysioTools Ltd

Exercise number 5.

Stand facing a wall. Press the wall with the toes of one leg holding the heel down.

Bring your hip forwards and feel the tension in the calf. Hold the position for 20 sec.

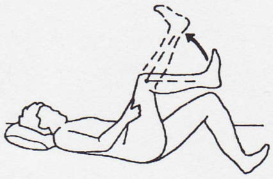

Exercise number 6.

Lie on your back with one knee bent. Bring the leg you are training up with the knee slightly bent. Hold the thigh still and straighten the knee until you feel tension behind the thigh.

Hold the tension 10 - 20 sec.

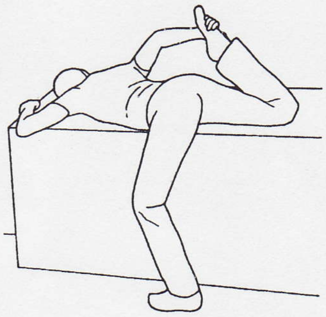

© PhysioTools Ltd

Exercise number 7.

Lie prone on a steady table with one leg on the floor.

Bend the knee of the leg lying on the table and pull the heel towards your buttock with your hand. Do not lift the upper part of your body. Hold 10 - 20 sec.

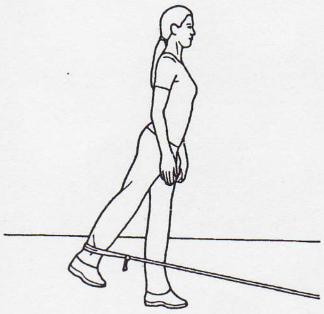

© PhysioTools Ltd

Exercise number 8.

Stand with the resistance band round your ankle. Fix the resistance band for example round the foot of a table.

Slide your foot backwards on the floor against the resistance of the band. You should be able to feel the tension in the back of your thigh and buttocks.

Do the exercise alternately.

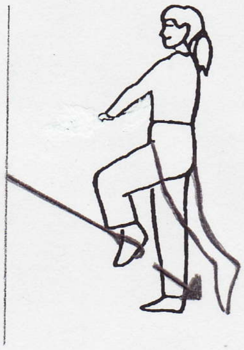

Exercise number 9.

Put the resistance band round your ankle. Push your leg down and diagonally backwards by straightening your knee against the resistance band. Do the exercise fast but carefully. The supporting leg should be slightly flexed, in a painfree position. You should be able to feel the tension in the back of your thigh and buttocks.

Do the exercise alternately.

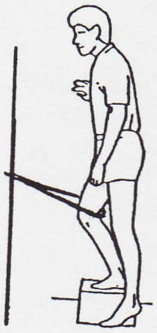

Exercise number 10.

Place the leg you are exercising on a small platform, letting the other leg hang outside the platform. Keep the knee of the leg your are exercising slightly flexed. Place the resistance band above your knee so that you can feel the resistance in the medial side of your thigh.

Straighten the knee and then slowly flex it again so that the foot outside the platform lightly touches the floor.

© PhysioTools Ltd

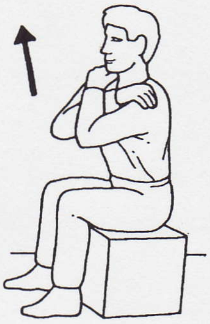

Exercise number 11.

Sit on a stool. Put your weight on both feet. Stand up by straightening both knees. Lower yourself slowly down on to the stool so that your buttocks slightly touch the stool and then stand up again.

Don´t let the knees go over the toe-line.

© PhysioTools Ltd

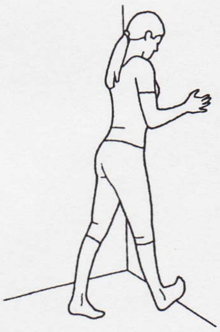

Exercise number 12.

Stand facing a wall. Press the wall with the toes of one leg holding the heel down.

Bring your hip forwards and feel the tension in the calf. Hold the position for 20 sec.

© PhysioTools Ltd

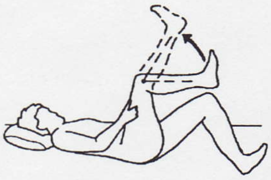

Exercise number 13.

Lie on your back with one knee bent. Bring the leg you are training up with the knee slightly bent. Hold the thigh still and straighten the knee until you feel tension behind the thigh.

Hold the tension 10 - 20 sec.

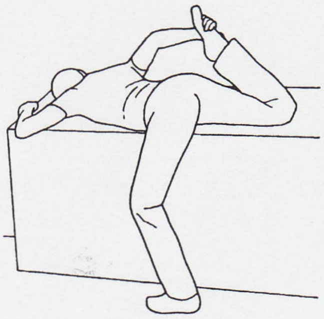

Exercise number 14.

Lie prone on a steady table with one leg on the floor.

Bend the knee of the leg lying on the table and pull the heel towards your buttock with your hand. Do not lift the upper part of your body. Hold 10 - 20 sec.

© PhysioTools Ltd
