# Supplementary material for: Knee arthroscopy and exercise versus exercise only for chronic patellofemoral pain syndrome: a randomized controlled trial
Source: BMC Med. 2007 Dec 13;5:38. doi: 10.1186/1741-7015-5-38 (PMC2249589; doi:10.1186/1741-7015-5-38)
Supplement: Additional File 2 — The Kujala score. [file 1741-7015-5-38-S2.pdf]

APPENDIX

ANTERIOR KNEE PAIN (Sheet code: __________________)

Name: ____ Date: _________________

Age: _________

Knee: L/R

Duration of symptoms: ______ years _______ months

For each question, circle the latest choice (letter), which corresponds to your knee symptoms.

1. Limp
(a) None (5)
(b) Slight or periodical (3)
(c) Constant (0)

2. Support
(a) Full support without pain (5)
(b) Painful (3)
(c) Weight bearing impossible (0)

3. Walking
(a) Unlimited (5)
(b) More than 2 km (3)
(c) 1-2 km (2)
(d) Unable (0)

4. Stairs
(a) No difficulty (10)
(b) Slight pain when descending (8)
(c) Pain both when descending and ascending (5)
(d) Unable (0)

5. Squatting
(a) No difficulty (5)
(b) Repeated squatting painful (4)
(c) Painful each time (3)
(d) Possible with partial weight bearing (2)
(e) Unable (0)

6. Running
(a) No difficulty (10)
(b) Pain after more than 2 km (8)
(c) Slight pain from start (6)
(d) Severe pain (3)
(e) Unable (0)

7. Jumping
(a) No difficulty (10)
(b) Slight difficulty (7)
(c) Constant pain (2)
(d) Unable (0)

8. Prolonged sitting with the knees flexed
(a) No difficulty (10)
(b) Pain after exercise (8)
(c) Constant pain (6)
(d) Pain forces to extend knees temporarily (4)
(e) Unable (0)

9. Pain
(a) None (10)
(b) Slight and occasional (8)
(c) Interferes with sleep (6)
(d) Occasionally severe (3)
(e) Constant and severe (0)

10. Swelling
(a) None (10)
(b) After severe exertion (8)
(c) After daily activities (6)
(d) Every evening (4)
(e) Constant (0)

11. Abnormal painful kneecap (patellar) movements (subluxations)
(a) None (10)
(b) Occasionally in sports activities (6)
(c) Occasionally in daily activities (4)
(d) At least one documented dislocation (2)
(e) More than two dislocations (0)

12. Atrophy of thigh
(a) None (5)
(b) Slight (3)
(c) Severe (0)

13. Flexion deficiency
(a) None (5)
(b) Slight (3)
(c) Severe (0)

Reference: Kujala UM, Jaakkola LH, Koskinen SK, Taimela S, Hurme M, Nelimarkka O: **Scoring of patellofemoral disorders.** *Arthroscopy* 1993, **9:**159-163.
